# Crystallization of Lithium Zeolites onto Foamed Geopolymers

**DOI:** 10.3390/ma18235310

**Published:** 2025-11-25

**Authors:** Carlo Gravino, Assunta Campanile, Claudio Ferone, Domenico Caputo, Nicola Gargiulo, Barbara Liguori, Paolo Aprea

**Affiliations:** 1 ACLabs—Department of Chemical, Materials and Production Engineering, University of Naples Federico II, 80125 Naples, Italy; carlo.gravino@unina.it (C.G.); assunta.campanile@unina.it (A.C.); domenico.caputo@unina.it (D.C.); barbara.liguori@unina.it (B.L.); 2 Department of Engineering, University of Naples Parthenope, 80143 Naples, Italy; claudio.ferone@uniparthenope.it; 3 National Interuniversity Consortium of Materials Science and Technology (INSTM), 50121 Firenze, Italy; 4 CeSMA—Advanced Metrological and Technological Services Center, University of Naples Federico II, 80125 Naples, Italy; ngargiul@unina.it

**Keywords:** geopolymers, ceramic, foams, zeolite nucleation, lithium-containing zeolites

## Abstract

Lithium-containing zeolites are receiving significant attention due to their intriguing properties in various industrial applications, mainly related to gas separation and catalyzed processes. This paper presents an in-depth exploration of a nucleation strategy aimed at producing porous ceramic monoliths enriched with lithium zeolites. The synthesis was obtained by means of a geopolymer gel conversion, carried out by submerging either sodium- or lithium-rich geopolymers in lithium hydroxide solutions and performing a hydrothermal treatment. A full factorial design of experiments (DoE) was adopted to investigate the effect of LiOH molarity, treatment temperature, and time on the zeolite content in the samples. The most abundant and recurring zeolites obtained were Li-ABW (ABW) and lithium edingtonite (EDI). Concerning the lithium/sodium-containing systems, the competing presence of sodium directed the nucleation towards faujasite as well, together with minor amounts of other zeolites. In contrast, in pure lithium treatment media, the samples showed just ABW and EDI as the only crystalline phases.

## 1. Introduction

Zeolites and geopolymers, also known as aluminosilicate inorganic polymers, are two classes of materials sharing the same chemistry but different structural features: while zeolites are crystalline, nanoporous materials, geopolymers are amorphous [1]. These peculiarities provide them with different properties: zeolites are widely used as ion exchangers, adsorbents, and catalysts, while geopolymers are mainly studied for their good mechanical properties that, together with the low energy and ease of production, make them a feasible alternative to structural materials like cement [2,3]. From a structural point of view, both materials are based on connected tetrahedra containing oxygen at the vertexes and an atom of silicon or aluminum at the center. For this reason, geopolymers are often considered as the amorphous counterpart of crystalline zeolites. Since a significant fraction of the solid produced during geopolymer formation likely consists of nanometer-sized crystalline structures that resemble zeolites nuclei [1,4] the possibility of triggering the growth of such structures into zeolite crystals is extremely appealing, because it will eventually lead to the obtainment of zeolite-enriched monoliths.

In recent years, novel strategies to obtain shaped zeolite monoliths through the growth of zeolite crystals from geopolymer precursors have been proposed [3,5,6,7,8]. Since the nucleation and growth of the zeolite crystals are affected by the conditions of the reaction system (temperature, pressure, time, Si/Al ratio, nature and concentration of the alkali activating solution), different types of zeolites have been produced by varying these parameters [9,10,11,12,13]. Scientific research about zeolite–geopolymer hybrids is increasingly growing, since these materials exhibit properties that merge those of their constituents: while zeolites have a large surface area and functional abilities, geopolymers provide them with a strong and stable support [14]. This makes the hybrid composites an interesting alternative to the conventional pellets used in several industrial processes, which are obtained from zeolite powders by means of treatments like pelletization, granulation, or extrusion, that often limit the performance of the active material. On the contrary, the production of zeolite-rich composites by geopolymer conversion appears to bring several advantages related to ease and low cost of the process, mechanical strength of the composite (given by the geopolymeric backbone), and active functionality (provided by the zeolites).

Recently, a further evolution of this approach has been proposed with the aim of improving the performance of zeolite–geopolymer composites by producing porous monoliths, in turn promoting a better diffusion inside the active material. To obtain a zeolite-rich porous monolith a suitable foaming agent and/or a surfactant is used during the production process [15,16]. This approach allowed the authors to produce LTA- and FAU-rich monoliths, successfully tested in water softening processes [17].

This paper focuses on a further exploration of the proposed foaming and nucleation strategy aimed at producing porous monoliths enriched in lithium zeolites. Lithium zeolites, like Li-ABW and Li-EDI, are paid great attention thanks to their interesting peculiarities for catalytic applications [18], removal of heavy metals and dyes from wastewaters [19,20], and CO_2_ separation [21,22] and capture [23,24]. Several papers propose synthesis routes to obtain lithium zeolites (mainly ABW) from different sources, ranging from halloysite [12] to waste, like fly ash [18,25,26]. However, these papers reported the production of ABW as powder, and only few papers report the synthesis through conversion of non-porous geopolymers [19,20,27]. To the best of our knowledge, there are still no studies focusing on the synthesis of lithium-containing zeolites in foamed systems. Therefore, in this paper, a systematic investigation of the synthesis conditions in Na-Li and Li systems have been carried out to control lithium zeolite nucleation in binder-free, foamed, monolithic lithium zeolites.

## 2. Experimental Section

### 2.1. Synthesis of the Foamed Zeolite-Rich Geopolymer

Zeolite-enriched geopolymeric monoliths were obtained by following the approach reported in [3,8]. Accordingly, the process was carried out in two steps, the first consisting in the production of a foamed geopolymer by means of an alkaline activated reaction, and the second, adopted to convert the geopolymer to zeolite, consisting in a post-synthesis hydrothermal treatment.

**Figure 1:**
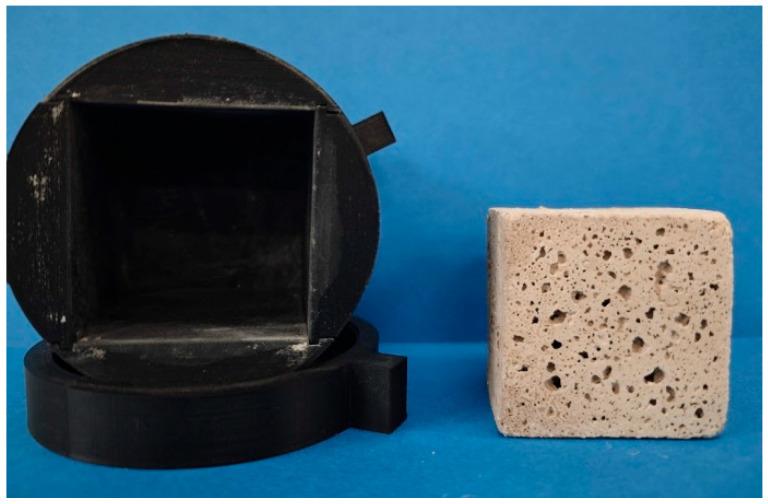
Foamed geopolymer after the consolidation in the mold.

For the synthesis process, metakaolin (IMCD Italia SpA, Milan, Italy, powder) was used as aluminosilicate source. Its chemical composition, reported by the vendor, was as follows: Al_2_O_3_ 41.90 wt%; SiO_2_ 52.90 wt%; K_2_O 0.77 wt%; Fe_2_O_3_ 1.60 wt%; TiO_2_ 1.80 wt%; MgO 0.19 wt%; CaO 0.17 wt%. The QPA analysis (See Figure S1 and Table S6) revealed an amorphous content of 73.89%. In addition, 10 M NaOH solution (Titolchimica, Villamarzana, Italy, 98% purity NaOH, pellets) was used as the alkaline-activating medium, and silicon (Riedel-de Haën, Milan, Italy, 99.9% purity, powder) as the foaming agent. To produce the foam, 10 g of metakaolin was carefully dry mixed with 0.1 g of silicon until achieving a uniform dispersion, and then 11 g of activating solution was added. The resulting slurry was quickly stirred, poured in a 5 cm-sized cubic mold (3D-printed in TPU) and cured in an oven at 40 °C for 24 h (Figure 1).

To convert the monoliths into a zeolite-rich foam, the samples were subjected to a hydrothermal conversion treatment with a LiOH solution (Merck, Milan, Italy, 98% purity) at different molarities. Accordingly, the foams were put in a PET container and submerged in the solution at a solid-to-liquid ratio equal to 60 g L^−1^ and kept at a certain temperature for a certain time under static conditions. After the treatment the samples were collected, thoroughly washed with distilled water and dried overnight at 60 °C. All the samples preserved their mechanical integrity and macroscopic morphology after hydrothermal treatment.

A full factorial design of experiments (DoE) was adopted to investigate the effect of the hydrothermal treatment temperature, time, and LiOH molarity on the zeolite content in the samples. In fact, the nucleation of zeolites inside a geopolymeric matrix is mainly induced by the temperature and the alkalinity of the solution, which promote the dissolution of the geopolymer. As soon as aluminate and silicate anions are released from the matrix, they start to interact with the cations in solution to precipitate zeolite crystals or to enlarge the nanosized nuclei present on the geopolymer surface. If the temperature and the molarity of the solution are wisely chosen, the rate of dissolution of the geopolymer matches that of growth of the zeolite crystals on the geopolymer matrix so that by the end of the treatment, the resulting solid is still mechanically coherent but enriched with zeolites. In this case the hydrothermal treatment induced a so-called pseudomorphic transformation [5]. Concerning the DoE table, each factor varied on three levels (treatment time: 3, 7, 21 days; treatment temperature: 40, 60, 80 °C; LiOH molarity: 0.25, 0.75, 1.5 M).

Based on the results from this experimental campaign, hereafter named “Na/Li route”, a second set of samples was produced, this time by using a 3 M LiOH solution instead of NaOH for the foam production. The samples obtained with the lithium alkaline solution were subjected to the same DoE experimental table. In the following, this second synthesis route will be referred to as “Li route”.

In total, 27 samples were produced for each geopolymer synthesis route, hereafter identified as “route-xT/yd/zM”, with route: “Li” or “Na/Li”, x: treatment temperature, y: treatment time, z: concentration of the LiOH solution used in the hydrothermal treatment. The investigated treatment conditions are reported in the Supplementary Material (Table S1). The robustness of the hydrothermal treatments has been checked on selected samples, and the variation in the obtained results was less than 7%.

### 2.2. Sample Characterization

The presence of zeolites in the samples was checked by means of X-Ray Diffraction using a Malvern Panalytical X’Pert Pro diffractometer (Malvern, Worcestershire, UK) equipped with PixCel 1D detector (radiation: CuK_α1_/K_α2_; operating conditions: 40 kV, 40 mA; scanning range: 5 to 80 °2θ; step size: 0.0131 °2θ; counting time: 40 s per step). Crystalline phases were identified with PANalytical HighScore software v. 5.3a, provided with the ICDD PDF-5+ database. To have a quick assessment of the abundance of each zeolite present in the samples, the integrated intensity of its main peak was used as a reference. This parameter was also used in the statistical analysis to express the response of the system in terms of the targeted zeolite yield (ABW or EDI). On selected samples, a quantitative phase analysis (XRD-QPA) was performed with the RIR/Rietveld method. Approximately 10% of NIST 676a standard (corundum) was added to the samples prior to be analyzed, as well as to estimate the amorphous content. The morphological features of the samples were assessed by acquiring SEM micrographs, using a Tescan Vega 3 equipped with the software “Tescan Essence”. Preliminarily, all samples were sputtered with gold. On selected samples, textural properties were investigated: open porosity was evaluated according to the European Standards UNI 11060:2003 [28], which reports methods for the determination of voids of natural and artificial stones, while the specific surface area was measured, either on the samples after the zeolite nucleation or on the pristine geopolymeric foams, by means of N_2_ adsorption/desorption. Tests were carried out using a volumetric apparatus (Micromeritics 3Flex, Bettola-Zeloforamagno, Italy) on samples previously degassed overnight at 200 °C in a nitrogen stream. The specific area (SSA) was evaluated by fitting the Brunauer–Emmett–Teller (BET) model to the experimental data. Moreover, mechanical characterization was performed on selected specimens using a Tensometer 2020 device by Alpha Technologies (Milan, Italy) with a 5 or 10 kN load cell and a crossbar lowering speed of 2 mm min^−1^.

### 2.3. Response Analysis and Model Fitting

The influence of the three factors (molarity, temperature, and time) on the yield of the target zeolites, ABW and EDI, has been assessed by performing the analysis of variance (ANOVA) on the set of integrated intensities of each phase. To fit the experimental data, a model was used to express the response (in terms of integrated intensity of the main peak of the target phase) as a function of the three factors and their mutual interactions, as symbolically shown in Equation (1):

(1) TARGET ZEOLITE≈M+T+t+M:T+M:t+T:t

where M, T, and t stand for the effect of molarity, temperature and time, and M:T, M:t, and T:t represent their mutual interactions.

By performing the ANOVA, a model term can be considered statistically significant if its *p*-value is lower than <0.05.

## 3. Results and Discussion

### 3.1. Na/Li-Route

**Figure 2:**
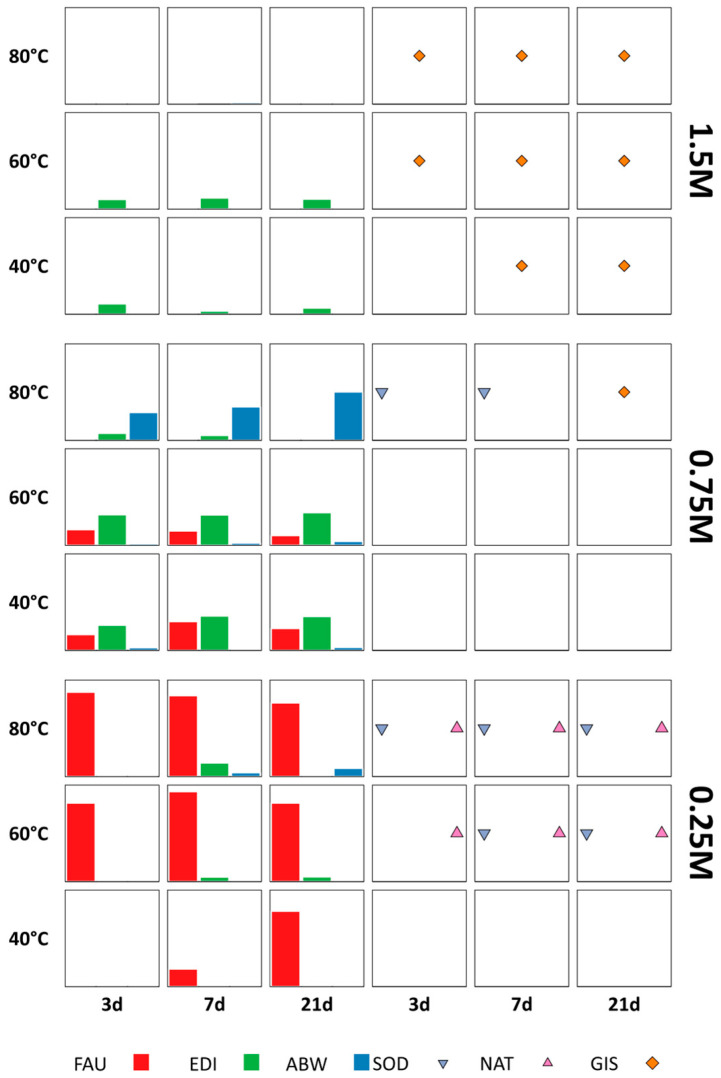
Occurrence map of the zeolites detected in the samples obtained by following the Na/Li route. Plots on the left side show the amount of the most abundant phases, expressed as the integrated intensity of the most intense peak (counts∙°2θ). The presence of other zeolites in traces is also indicated (plots on the right side).

A detailed map of the diffraction pattern of every sample, as a function of molarity, treatment time and temperature, is reported in Figures S2–S4. The XRD analysis showed that at the lowest temperature investigated (40 °C) the crystallization was poor, independently of the molarity and duration of the treatment. The same occurred at the highest molarity (1.50 M), independently of time and temperature. Moreover, besides Li-ABW (ABW) and lithium edingtonite (EDI), faujasite (FAU) was detected in many of the samples produced at the lowest molarity. Traces of several other zeolites, like natrolite (NAT), sodalite (SOD), and gismondine (GIS), were also found in many samples. The presence of these phases was just indicated, but the integrated intensity of their main peak was not measured, because of their very low signal. The nucleation of such a broad set of spurious phases is likely due to the presence of a high amount of sodium, remaining from the activating solution employed to produce the geopolymeric foam. Moreover, smaller amounts of calcium, potassium, and magnesium were also present in the metakaolin. The results are summarized in Figure 2, where all the obtained zeolites are reported as a function of the LiOH molarity, temperature, and time of the hydrothermal treatment.

#### 3.1.1. Occurrence of ABW

**Figure 3:**
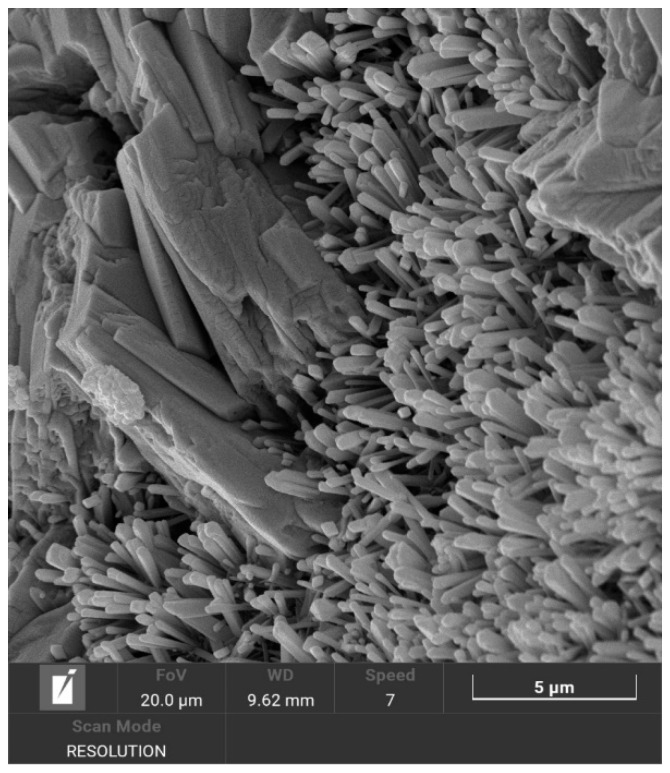
SEM image of the sample Na/Li-80 °C/21 d/0.75 M showing ABW as rod-like/fibrous crystals.

Figure 2 illustrates how alkalinity is the most influential parameter on the occurrence of ABW, followed by the temperature, while time does not appear to particularly determine the ABW yield. As can be noted, in the samples treated with 0.25 M LiOH ABW was detectable only at 80 °C, after 7 and 21 days, in form of rod-like crystals (Figure 3). This suggests a higher activation energy and/or a longer induction period with respect to the nucleation of faujasite, which was the predominant phase obtained at that molarity. The presence of ABW was also detected in every sample treated with 0.75 M LiOH, with an amount roughly increasing with the treatment temperature and/or time: in the Na/Li-80 °C/21 d/0.75 M sample, ABW was the only zeolite detected, with an amount of about 77% (see Figure S5 and Table S6). Apparently, the higher value of the alkalinity of the solution increased the dissolution rate of the geopolymeric matrix, improving the ABW yield. On the contrary, a further increase in the LiOH concentration (1.5 M) negatively affected the synthesis process: small traces of ABW were only present at the highest treatment temperature and the longest treatment time. On the Na/Li-80 °C/21 d/0.75 M sample an assessment of structural integrity was also performed by using a Tensometer 2020 device by Alpha Technologies with a 5 or 10 kN load cell and a crossbar lowering speed of 2 mm min^−1^. The sample reported a compressive strength around 0.3 ± 0.04 MPa in accordance with data reported in Campanile et al. [16] regarding foams obtained in sodium media. Concerning the textural properties, the open porosity turned out to be 69.2%, while the surface area was equal to 21.09 ± 0.15 m^2^ g^−1^, considerably higher than the surface area of the same geopolymer before the hydrothermal treatment (7.34 ± 0.37 m^2^ g^−1^).

The analysis of the effect of the three factors, shown in Figure S6, confirmed that temperature and alkalinity have the most significant effect on the presence of such zeolite. The analysis of variance (ANOVA), reported in Table S2, highlighted that only molarity, temperature, and their interaction are significant model terms.

#### 3.1.2. Occurrence of Edingtonite

**Figure 4:**
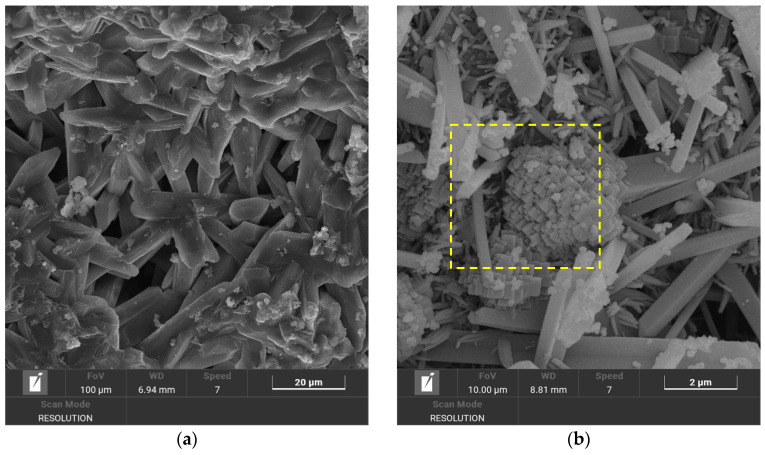
SEM images showing the different morphology of EDI crystals produced under different treatment conditions: (**a**) Na/Li-40 °C/21 d/1.5 M (EDI is present as acicular crystals), (**b**) Na/Li-80 °C/7 d/0.75 M (EDI crystals, highlighted in the yellow frame, are cubic).

The occurrence of edingtonite showed a different stability field: While EDI was never detected alone in none of the investigated samples, at 0.25 M, it was present in very small traces at 60 °C and 80 °C, after at least 7 days of treatment. At 0.75 M, it was present in higher amounts in every sample, except for that treated at 80 °C and 21 days. At 1.5 M, small amounts of EDI were present in the samples treated at 40 °C and 60 °C. This evidence suggests that temperature and molarity can have a counteracting effect on the zeolite nucleation. Interestingly, the crystals nucleated at low temperature and high molarity showed an acicular morphology, instead of the more common cubic one shown by the crystals nucleated at high temperature and intermediate molarity (Figure 4).

The effects plots (Figure S7) showed a significant influence of the temperature, as well as a less important effect of both temperature and time, consistently with the results of the ANOVA (Table S3).

Based on the results obtained from the Na/Li route, a new round of experiments has been performed by replacing the sodium hydroxide solution used for the preparation of the foams with a LiOH solution, so as to narrow down the cation pool in the treatment medium and, in turn, reduce the possibility of spurious nucleation. Due to the lower solubility of LiOH in water, a nearly saturated (3 M) solution was prepared.

### 3.2. Li-Route

**Figure 5:**
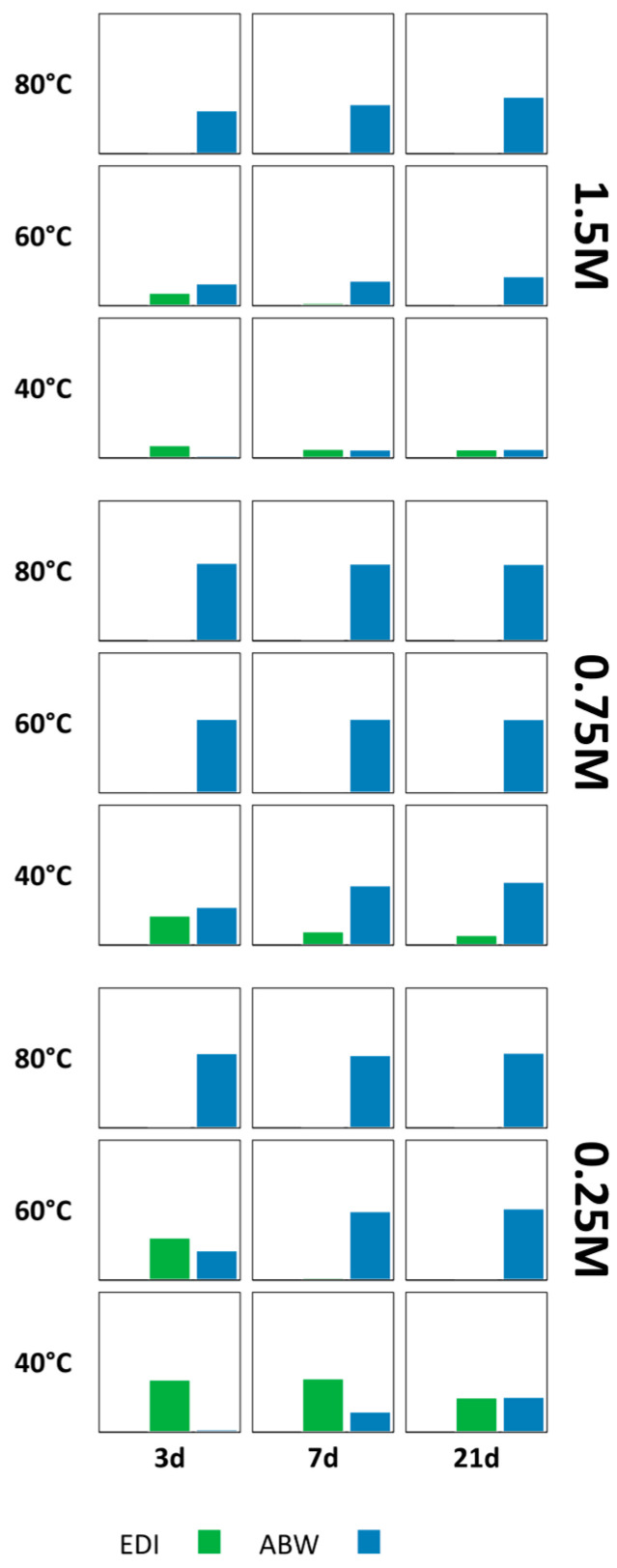
Occurrence map of the zeolites detected in the samples obtained by following the Li route. Plots show the amount of the most abundant phases, expressed as the integrated intensity of the most intense peak (counts∙°2θ).

As expected, the number of zeolites present in all the samples investigated were reduced only to EDI and ABW. Interestingly, the crystallization degree significantly increased: even the samples treated with a 0.25 M LiOH solution at the lowest temperature (40 °C) showed a noticeable presence of zeolites. Figure 5 summarizes the obtained results, while a detailed map of the diffraction patterns of each sample as a function of the treatment parameters is reported in Figures S8–S10.

#### 3.2.1. Occurrence of ABW

**Figure 6:**
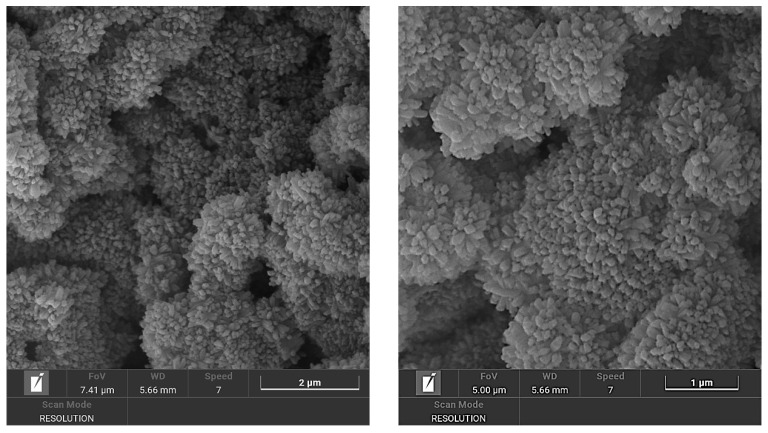
SEM image of the sample Li-60 °C/3 d/0.75 M. ABW is clearly visible as a uniform layer of sub-micrometric crystals.

ABW was detected at each of the investigated molarities, and its amount increased with the temperature, while being relatively independent of the treatment time. In general, all the samples showed the zeolite as a homogeneous “meadow” of bush-like assembled crystals (Figure 6). At the highest temperature (80 °C), it was the only phase present at every molarity and treatment time investigated. The highest observed amount, in terms of the integrated intensity of the most intense peak, has been found for the samples obtained at 80 °C and 0.75 M, but it has to be noted that, especially for this zeolite, the anisotropic crystal growth can affect the relative intensities of the peaks, thus making this assessment less precise. In particular, the sample most rich in ABW turned out to be the Li-80 °C/7 d/0.75 M, corresponding to an amount, estimated from the QPA, of 79.97% (see Figure S11 and Table S6). The compression strength of this sample, measured as described above, was comparable with that of the analogous sample produced through the Na/Li route, confirming a fair structural integrity. As per the textural properties, the open porosity turned out to be 63.4%, indicating that the different alkaline solution did not significantly affect the foaming process. Moreover, the surface area, equal to 19.81 ± 0.14 m^2^ g^−1^ in the foam before the treatment, rose to 34.16 ± 0.14 m^2^ g^−1^ in the ABW-containing sample.

The ANOVA results, reported in Table S4, pointed out that in this case the three factors were all statistically significant, while there was no interaction between them.

#### 3.2.2. Occurrence of Edingtonite

**Figure 7:**
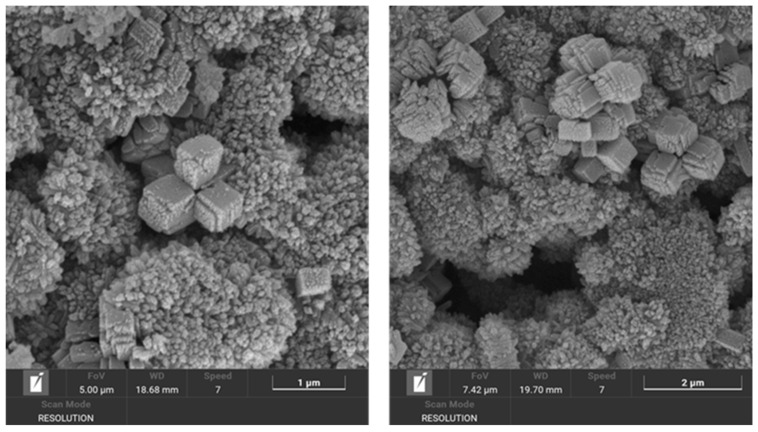
SEM image of the sample Li-60 °C/3 d/0.25 M. EDI is clearly visible as clusters of micron-sized cubic crystals.

As for the previous route, edingtonite was never found alone in the treated samples. It appeared in form of clusters of micron-sized cubic crystals (Figure 7), in accordance with the literature [29]. It had a significant presence, especially in the foams treated at the lowest investigated molarity (0.25 M). At 60 °C, EDI was clearly detectable after 3 days, became just an impurity after 7 days, and disappeared after 21 days of treatment. No EDI was identified in the samples treated at 80 °C. At 0.75 M edingtonite was still present, but only at 40 °C. Finally, at 1.50 M, low amounts of EDI were detected only at 40 °C and 60 °C.

Finally, from the ANOVA analysis (Table S5) it emerged that, once again, molarity, temperature, and their interaction were the only statistically significant model terms.

## 4. Final Discussion and Conclusions

In this work, for the first time, the nucleation of lithium zeolites from geopolymer foams under hydrothermal treatment in sodium/lithium-containing systems has been obtained. Concerning the foams produced by following the Na/Li route it emerged that ABW was dominant at medium-high molarities, but only at higher temperatures and treatment time, while EDI was present at medium-high molarities, but up to intermediate treatment temperatures. The nucleation of FAU, together with the scarce crystallization observed in the samples treated at the highest investigated molarity, can be due to the presence of sodium in the treatment medium. Sodium, initially embedded in the geopolymeric foam produced by using NaOH solution, is released under the action of the LiOH solution, and acts as competitive structure directing agent with respect to lithium. It is plausible that, at low molarities, the dissolution rate of the geopolymer is so slow that the local conditions at the solid-to-liquid interface (especially in terms of sodium concentration) induce the nucleation of faujasite, with a kinetic mainly dictated by the temperature. Higher molarities, however, make the FAU framework (density = 13.3 T-atoms∙1000 Å^−3^) unstable, leading to the appearance of more compact phases: EDI (framework density = 16.3 T-atoms∙1000 Å^−3^) and ABW (framework density = 17.0 T-atoms∙1000 Å^−3^), in accordance with Ostwald’s law [30]. These zeolites are probably not present in high amounts under the highest molarities investigated because of the fast dissolution rate of the geopolymer, together with the presence of sodium, that ultimately induce the crystallization of the other detected zeolites and of non-zeolitic, semi-amorphous phases.

Concerning the results obtained for the samples produced by following the Li route, the different composition of the activating solution dramatically reduced the number of zeolites present in the treated foams. Regarding ABW, its stability field was greatly enlarged, being present in almost every sample, and being the dominant phase at 0.75 M and 1.50 M at the higher temperatures. It is interesting to note that, by removing the sodium from the synthesis medium, it was possible to obtain a sample enriched with the same (or even higher) amount of ABW under milder alkalinity conditions (0.75 M) and shorter treatment time (7 days) with respect to those used in the Na/Li route. EDI, on the contrary, was never detected alone, was present in a narrow range of temperatures and molarities, being converted into ABW for longer treatment times and higher temperatures.

Another outcome of this work is given by the possibility to control the cation content either of the geopolymer formulation or of the hydrothermal solution to induce the crystallization of specific zeolite terms, whose content can be optimized by controlling the temperature, alkalinity, and treatment time, paving the way for fitting the composition of the foams to the desired application.

## Data Availability

The original contributions presented in this study are included in the article/Supplementary Material. Further inquiries can be directed to the corresponding author.

## Supplementary Materials

The following supporting information can be downloaded at: https://www.mdpi.com/article/10.3390/ma18235310/s1.
