# Supplementary material for: Crystallization of Lithium Zeolites onto Foamed Geopolymers
Source: Materials (Basel). 2025 Nov 25;18(23):5310. doi: 10.3390/ma18235310 (PMC12692886; doi:10.3390/ma18235310)
Supplement: Supplementary file 1 [file materials-18-05310-s001.zip › materials-3942112-supplementary.pdf]

Crystallization of Lithium Zeolites onto Foamed Geopolymers

Carlo Gravino [1], Assunta Campanile [1], Claudio Ferone [2,3], Domenico Caputo [1,3], Nicola Gargiulo [4], Barbara Liguori [1,3] and Paolo Aprea [1,3,*]

[1]ACLabs—Department of Chemical, Materials and Production Engineering, University of Naples Federico II, 80125 Naples, Italy; carlo.gravino@unina.it (C.G.); assun-ta.campanile@unina.it (A.C.);

domenico.caputo@unina.it (D.C.); barbara.liguori@unina.it (B.L.)

[2]Department of Engineering, University of Naples Parthenope, 80143 Naples, Italy;

claudio.ferone@uniparthenope.it

[3]National Interuniversity Consortium of Materials Science and Technology (INSTM), 50121 Firenze, Italy

[4]CeSMA—Advanced Metrological and Technological Services Center, University of Naples Federico II,

80125 Naples, Italy; ngargiul@unina.it

[*]Correspondence: paolo.aprea@unina.it

Table S1 - Treatment conditions for all the samples investigated, and main zeolites detected.

Label	route	T, °C	t, days	LiOH, M	EDI	ABW	FAU
Na/Li-40°C/3d/0.25M	Na/Li	40	3	0.25			
Na/Li-40°C/3d/0.75M	Na/Li	40	3	0.75	x	x	x
Na/Li-40°C/3d/1.5M	Na/Li	40	3	1.5	x		
Na/Li-40°C/7d/0.25M	Na/Li	40	7	0.25			x
Na/Li-40°C/7d/0.75M	Na/Li	40	7	0.75	x	x	x
Na/Li-40°C/7d/1.5M	Na/Li	40	7	1.5	x		
Na/Li-40°C/21d/0.25M	Na/Li	40	21	0.25			x
Na/Li-40°C/21d/0.75M	Na/Li	40	21	0.75	x	x	x
Na/Li-40°C/21d/1.5M	Na/Li	40	21	1.5	x		
Na/Li-60°C/3d/0.25M	Na/Li	60	3	0.25			x
Na/Li-60°C/3d/0.75M	Na/Li	60	3	0.75	x	x	x
Na/Li-60°C/3d/1.5M	Na/Li	60	3	1.5	x		
Na/Li-60°C/7d/0.25M	Na/Li	60	7	0.25	x		x
Na/Li-60°C/7d/0.75M	Na/Li	60	7	0.75	x	x	x
Na/Li-60°C/7d/1.5M	Na/Li	60	7	1.5	x		
Na/Li-60°C/21d/0.25M	Na/Li	60	21	0.25	x		x
Na/Li-60°C/21d/0.75M	Na/Li	60	21	0.75	x	x	x
Na/Li-60°C/21d/1.5M	Na/Li	60	21	1.5	x		
Na/Li-80°C/3d/0.25M	Na/Li	80	3	0.25			x
Na/Li-80°C/3d/0.75M	Na/Li	80	3	0.75	x	x	
Na/Li-80°C/3d/1.5M	Na/Li	80	3	1.5			
Na/Li-80°C/7d/0.25M	Na/Li	80	7	0.25	x	x	x
Na/Li-80°C/7d/0.75M	Na/Li	80	7	0.75	x	x	
Na/Li-80°C/7d/1.5M	Na/Li	80	7	1.5		x	

Na/Li-80°C/21d/0.25M	Na/Li	80	21	0.25		x	x
Na/Li-80°C/21d/0.75M	Na/Li	80	21	0.75		x	
Na/Li-80°C/21d/1.5M	Na/Li	80	21	1.5		x	
Li-40°C/3d/0.25M	Li	40	3	0.25	x	x	
Li-40°C/3d/0.75M	Li	40	3	0.75	x	x	
Li-40°C/3d/1.5M	Li	40	3	1.5	x	x	
Li-40°C/7d/0.25M	Li	40	7	0.25	x	x	
Li-40°C/7d/0.75M	Li	40	7	0.75	x	x	
Li-40°C/7d/1.5M	Li	40	7	1.5	x	x	
Li-40°C/21d/0.25M	Li	40	21	0.25	x	x	
Li-40°C/21d/0.75M	Li	40	21	0.75	x	x	
Li-40°C/21d/1.5M	Li	40	21	1.5	x	x	
Li-60°C/3d/0.25M	Li	60	3	0.25	x	x	
Li-60°C/3d/0.75M	Li	60	3	0.75		x	
Li-60°C/3d/1.5M	Li	60	3	1.5	x	x	
Li-60°C/7d/0.25M	Li	60	7	0.25	x	x	
Li-60°C/7d/0.75M	Li	60	7	0.75		x	
Li-60°C/7d/1.5M	Li	60	7	1.5	x	x	
Li-60°C/21d/0.25M	Li	60	21	0.25		x	
Li-60°C/21d/0.75M	Li	60	21	0.75		x	
Li-60°C/21d/1.5M	Li	60	21	1.5		x	
Li-80°C/3d/0.25M	Li	80	3	0.25		x	
Li-80°C/3d/0.75M	Li	80	3	0.75		x	
Li-80°C/3d/1.5M	Li	80	3	1.5		x	
Li-80°C/7d/0.25M	Li	80	7	0.25		x	
Li-80°C/7d/0.75M	Li	80	7	0.75		x	
Li-80°C/7d/1.5M	Li	80	7	1.5		x	
Li-80°C/21d/0.25M	Li	80	21	0.25		x	
Li-80°C/21d/0.75M	Li	80	21	0.75		x	
Li-80°C/21d/1.5M	Li	80	21	1.5		x	

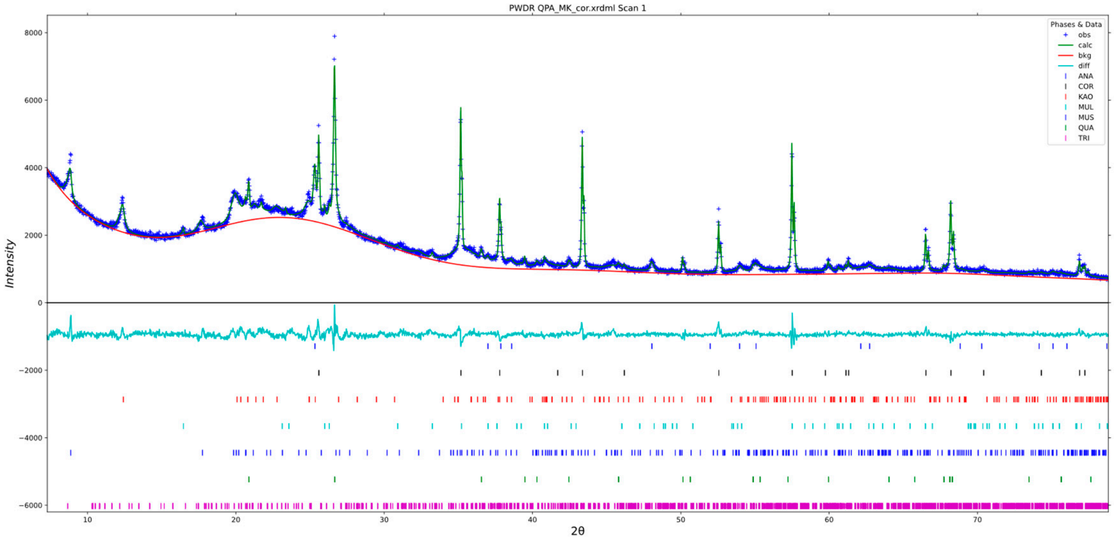

Figure S1 – Quantitative phase analysis of the metakaolin used for the foam synthesis, performed with the RIR/Rietveld method

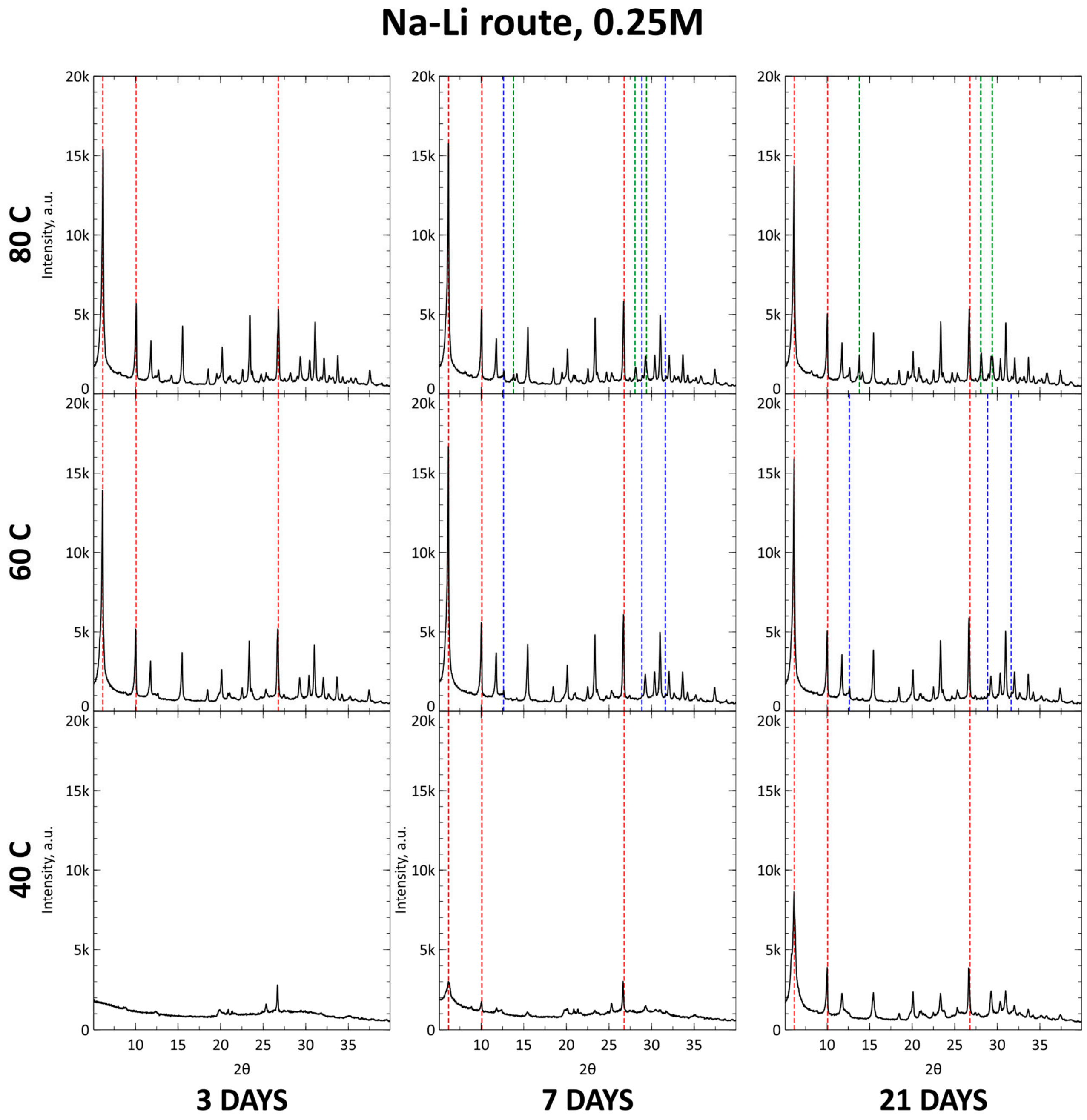

Figure S2 – XRD patterns of the sample obtained by following the "Na-Li route" with a 0.25M LiOH solution. Dashed lines indicate the three most intense peaks of the most abundant zeolites (Red: Li-FAU; BLUE: Li-EDI; GREEN: Li-ABW)

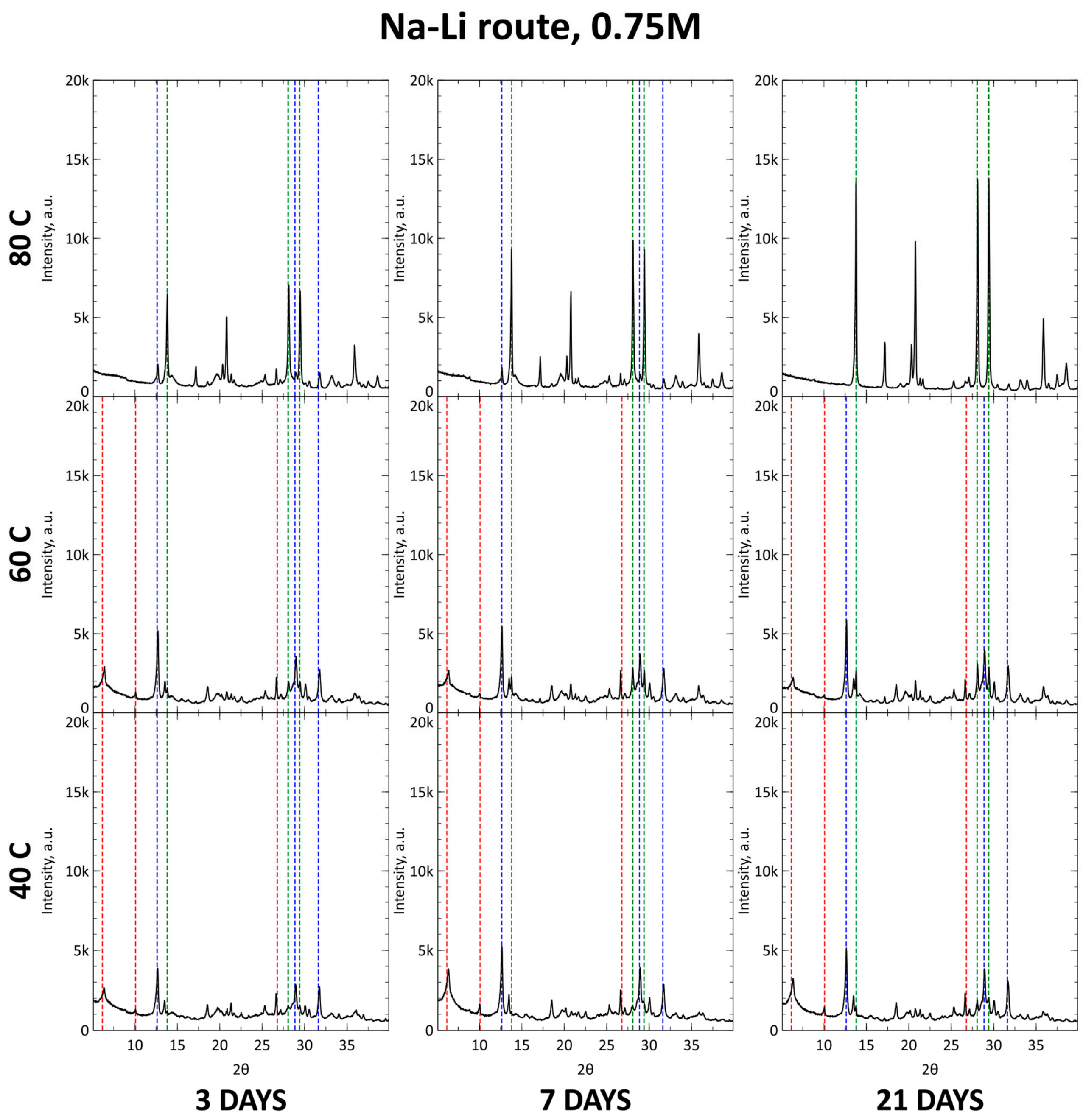

Figure S3 – XRD patterns of the sample obtained by following the "Na-Li route" with a 0.75M LiOH solution. Dashed lines indicate the three most intense peaks of the most abundant zeolites (Red: Li-FAU; BLUE: Li-EDI; GREEN: Li-ABW)

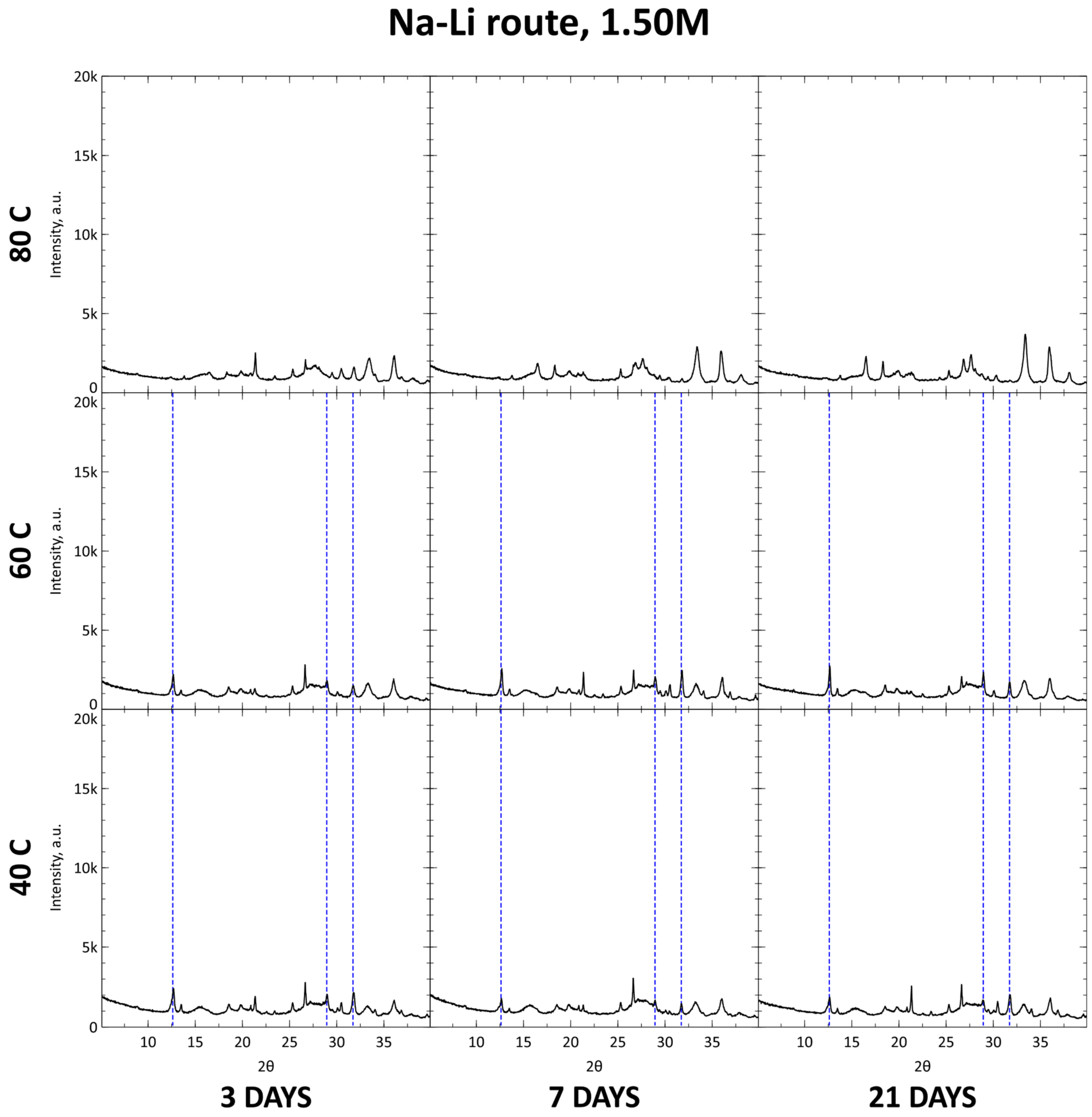

Figure S4 – XRD patterns of the sample obtained by following the "Na-Li route" with a 1.50M LiOH solution. Dashed lines indicate the three most intense peaks of the most abundant zeolites (Red: Li-FAU; BLUE: Li-EDI; GREEN: Li-ABW)

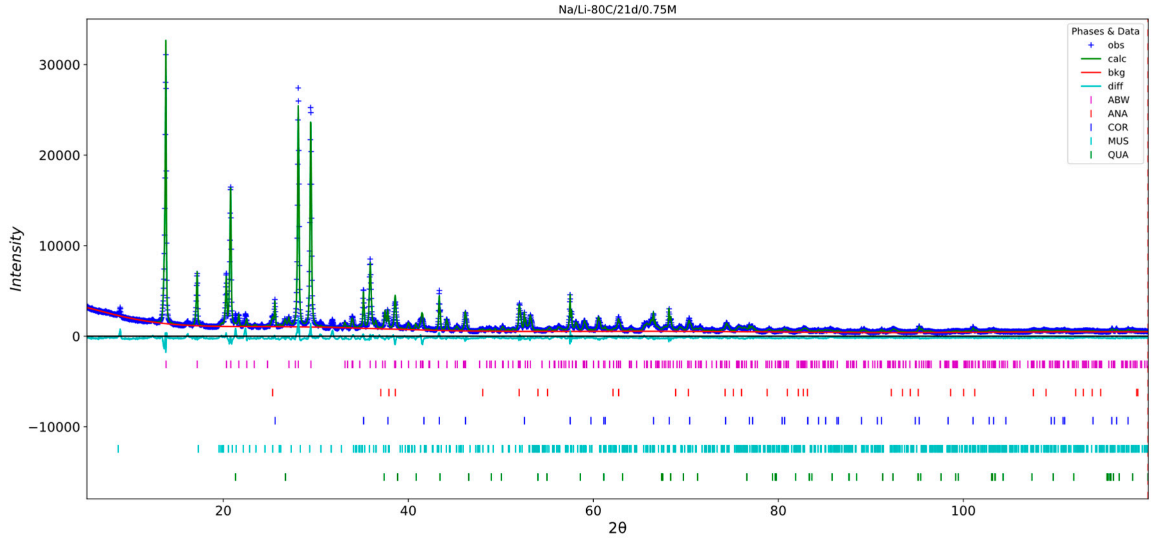

Figure S5 – Quantitative phase analysis of the sample Na/Li-/80°C/21d/0.75M, performed with the RIR/Rietveld method

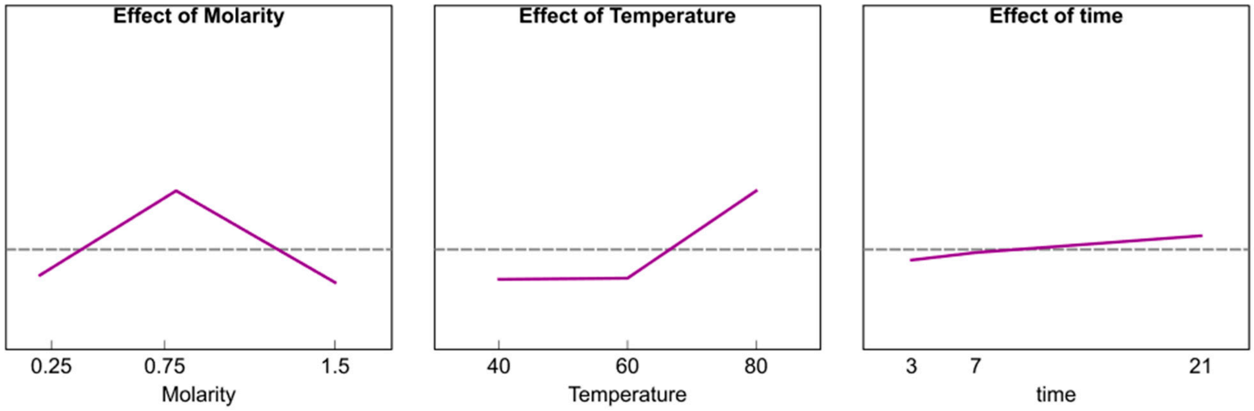

Figure S6 - Effect of molarity, temperature and time on the nucleation of ABW zeolite in the Na/Li system. Plots report the integrated intensity of the main peak of ABW averaged on level of each factor versus the factor levels. Dashed lines represent the overall average of all the samples.

Table S2. Analysis of variance of the ABW yield in Na/Li system

Model term	Degrees of freedom	Sum of Squares	Mean Square	F-value	p-value	Significant
M	3	2.31E+06	769103.7	74.33521	0.000003	yes
T	2	1.34E+06	671904.9	64.94078	0.000011	yes

**t**	2	8.05E+04	40257.81	3.890988	0.066026	no
**M:T**	4	1.77E+06	443743.1	42.88854	0.000019	yes
**M:t**	4	6.80E+04	17010.54	1.644098	0.254473	no
**T:t**	4	1.21E+05	30213.04	2.920142	0.092121	no

R^2 = 0.983; R^2_{adj} = 0.944

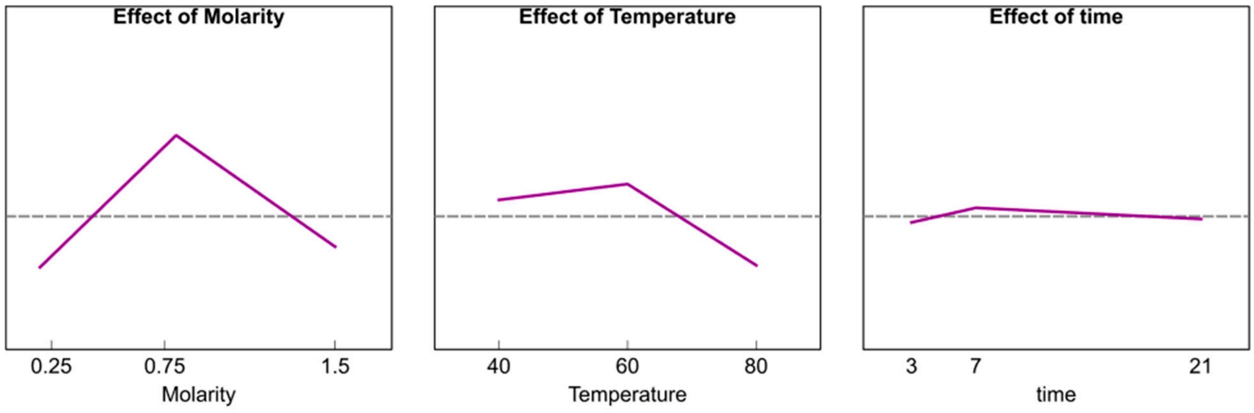

Figure S7 - Effect of molarity, temperature and time on the nucleation of EDI zeolite in the Na/Li system. Plots report the integrated intensity of the main peak of ABW averaged on level of each factor versus the factor levels. Dashed lines represent the overall average of all the samples.

Table S3. Analysis of variance of the EDI yield in Na/Li system

**Model term**	**Degrees of freedom**	**Sum of Squares**	**Mean Square**	**F-value**	**p-value**	**Significant**
**M**	3	6.29E+06	2.10E+06	99.61366	0.000001	yes
**T**	2	9.70E+05	4.85E+05	23.05128	0.000478	yes
**t**	2	3.05E+04	1.52E+04	0.724692	0.513743	no
**M:T**	4	1.24E+06	3.10E+05	14.73915	0.000922	yes
**M:t**	4	6.70E+04	1.68E+04	0.796658	0.559581	no
**T:t**	4	6.53E+04	1.63E+04	0.776389	0.570479	no

R^2 = 0.936; R^2_{adj} = 0.908

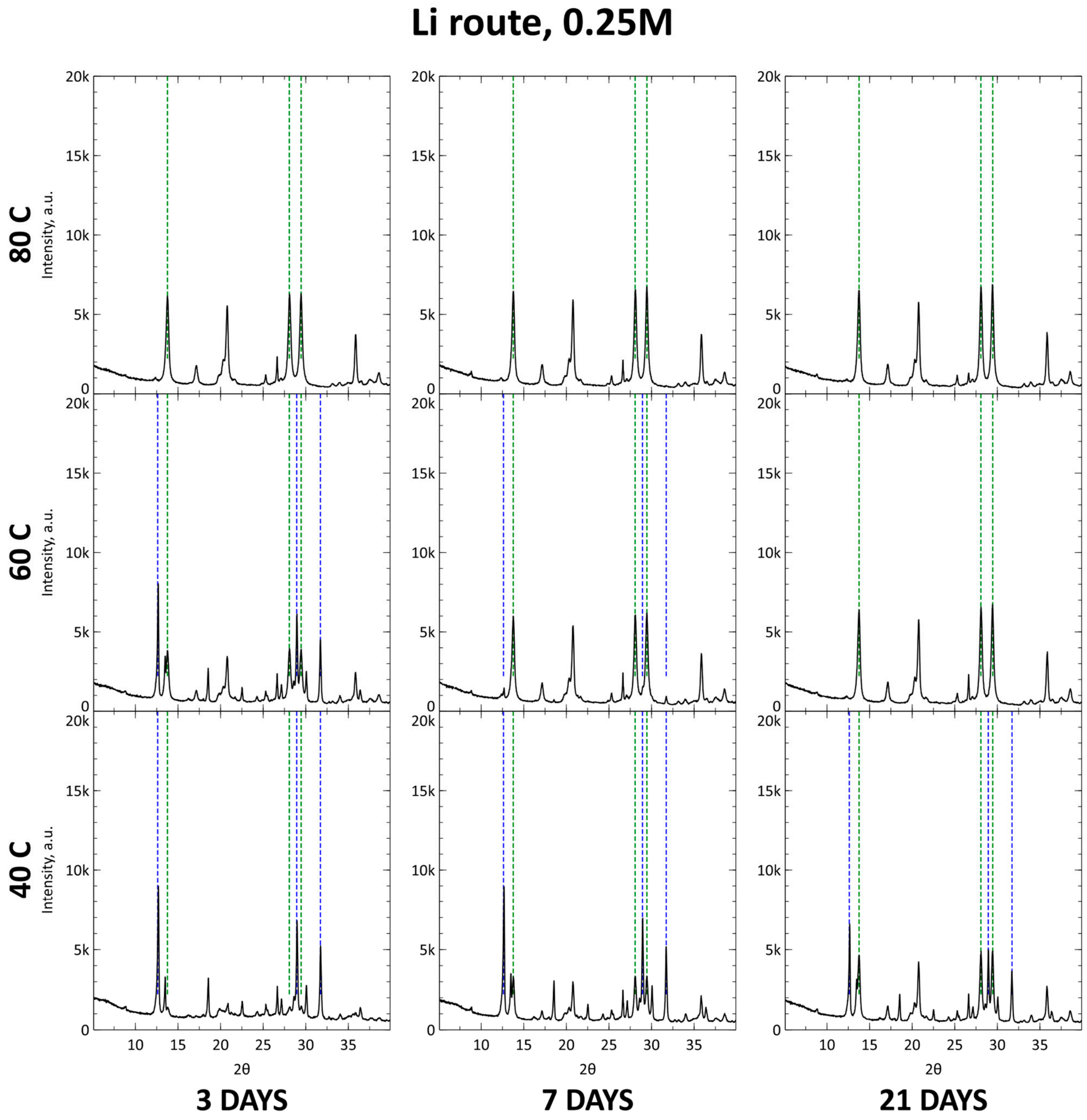

Figure S8 – XRD patterns of the sample obtained following the "Li route" with a 0.25M LiOH solution. Dashed lines indicate the three most intense peaks of the most abundant zeolites (BLUE: Li-EDI; GREEN: Li-ABW)

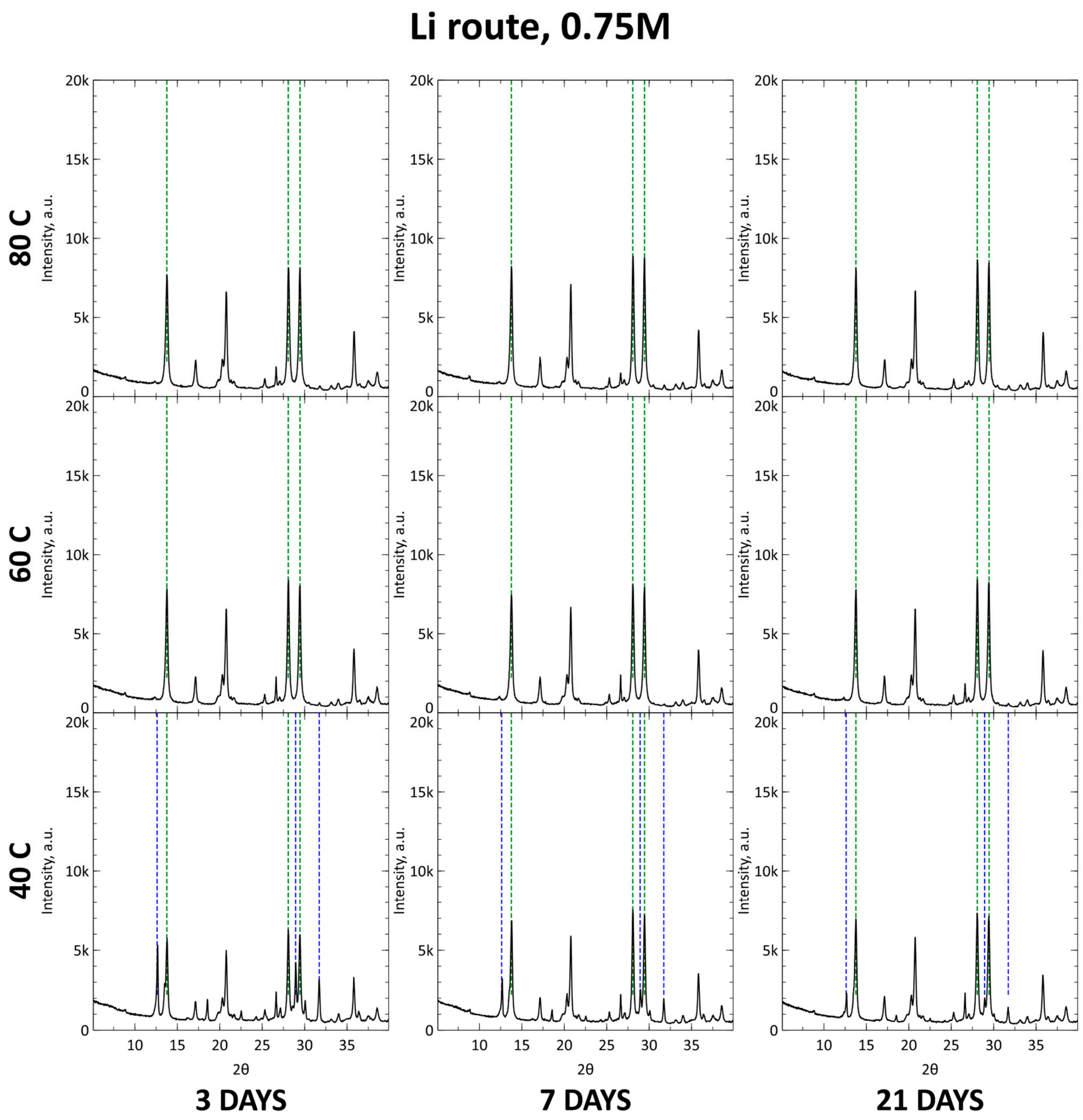

Figure S9 – XRD patterns of the sample obtained by following the "Li route" with a 0.75M LiOH solution. Dashed lines indicate the three most intense peaks of the most abundant zeolites (BLUE: Li-EDI; GREEN: Li-ABW)

Li route, 1.50M

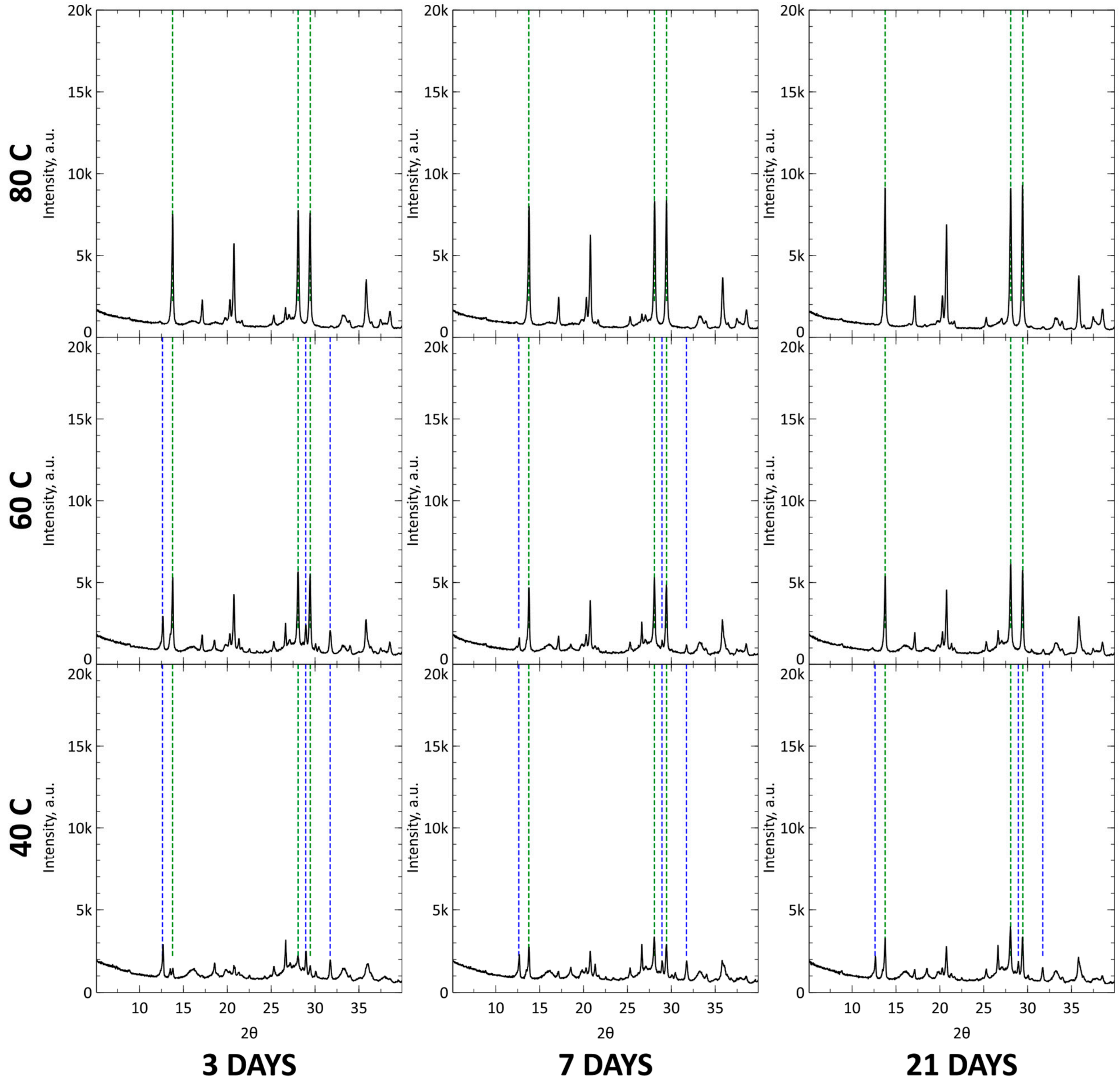

Figure S10 – XRD patterns of the sample obtained by following the "Li route" with a 1.50M LiOH solution. Dashed lines indicate the three most intense peaks of the most abundant zeolites (BLUE: Li-EDI; GREEN: Li-ABW)

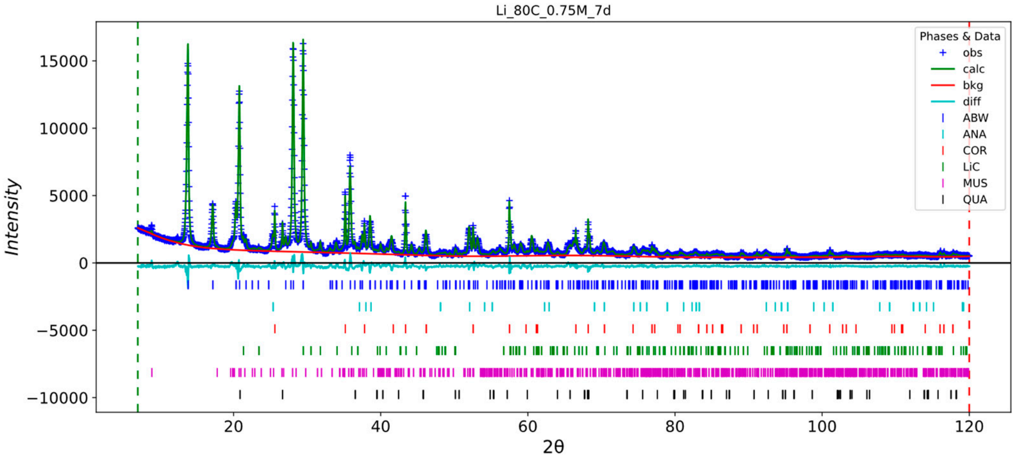

Figure S11 – *Quantitative phase analysis of the sample* Li-80/7d/0.75M, *performed with the RIR/Rietveld method*

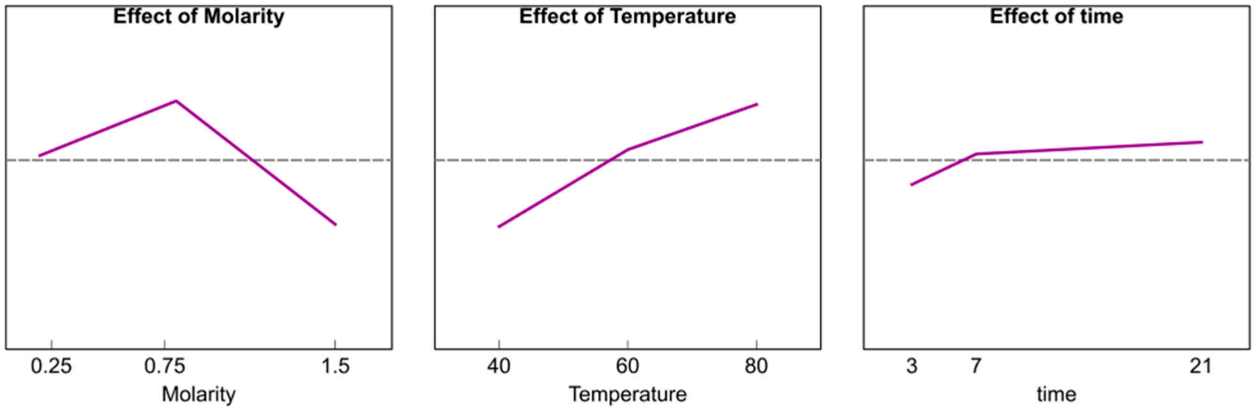

Figure S12 - Effect of molarity, temperature and time on the nucleation of ABW zeolite in the Li system. Plots report the integrated intensity of the main peak of ABW averaged on level of each factor versus the factor levels. Dashed lines represent the overall average of all the samples.

Table S4. Analysis of variance of the ABW yield in Li system

Model term	Degrees of freedom	Sum of Squares	Mean Square	F-value	p-value	Significant
M	3	4.49E+07	1.50E+07	306.8111	1.35E-08	yes
T	2	4.73E+06	2.37E+06	48.50864	3.37E-05	yes
t	2	5.91E+05	2.96E+05	6.057452	2.50E-02	yes
M:T	4	5.81E+05	1.45E+05	2.977941	8.84E-02	no
M:t	4	1.89E+05	4.72E+04	0.966754	4.76E-01	no
T:t	4	1.78E+05	4.45E+04	0.912351	5.01E-01	no

R^2 = 0.966; R^2_{adj} = 0.889

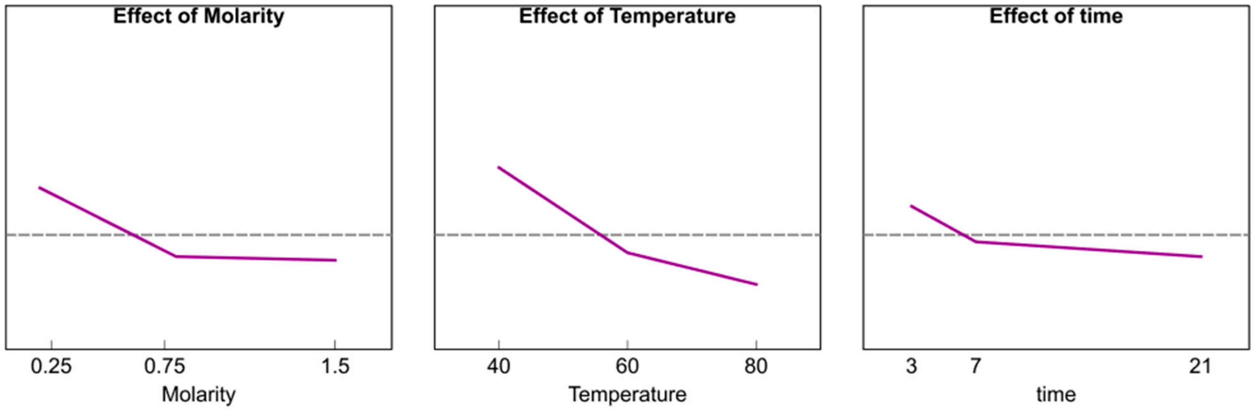

Figure S13 - Effect of molarity, temperature and time on the nucleation of EDI zeolite in the Li system. Plots report the integrated intensity of the main peak of ABW averaged on level of each factor versus the factor levels. Dashed lines represent the overall average of all the samples.

Table S5. Analysis of variance of the EDI yield in Li system

Model term	Degrees of freedom	Sum of Squares	Mean Square	F-value	p-value	Significant
**M**	3	2.78E+06	926316.3	20.59186	0.000405	yes
**T**	2	1.90E+06	951649.1	21.155	0.000639	yes
**t**	2	3.49E+05	174477.5	3.878605	0.066442	no
**M:T**	4	7.82E+05	195452.4	4.344875	0.036915	yes
**M:t**	4	1.24E+05	31016.09	0.689483	0.619393	no
**T:t**	4	2.28E+05	57011.48	1.267356	0.358209	no

R^2 = 0.922; R^2_{adj} = 0.746

Table S6 – Identified phases and relative weight percentage of the metakaolin and selected samples.

Phase	METAKAOLIN	Na/Li-80C/21d/0.75M	Li-80C/7d/0.75M
ABW	-	76.95	79.97
Anatase	1.28	0.96	1.39
Kaolinite	8.92	-	-
Mullite	1.41	-	-
Muscovite	10.61	1.13	4.56
Quartz	3.52	0.81	1.03
Trydimite	0.36	-	-
Lithium carbonate			3.44
Amorphous	73.89	20.15	9.61
**wR%***	3.91	7.99	6.64

** wR% = weighed profile residual, indicates the goodness of fit (the lower the value, the better the fit)*
